# Offset-FA: A Uniform Method to Handle Both Unbounded and Bounded Repetitions in Regular Expression Matching

**DOI:** 10.3390/s22207781

**Published:** 2022-10-13

**Authors:** Chengcheng Xu, Kun Yu, Xinghua Xu, Xianqiang Bao, Songbing Wu, Baokang Zhao

**Affiliations:** 1 National Key Laboratory of Science and Technology on Vessel Integrated Power System, Naval University of Engineering, Wuhan 430033, China; 2 College of Computer, National University of Defense Technology, Changsha 410073, China

**Keywords:** CPS security, deep-packet inspection, regular expression matching, state explosion

## Abstract

With the exponential growth of cyber–physical systems (CPSs), security challenges have emerged; attacks on critical infrastructure could result in catastrophic consequences. Intrusion detection is the foundation for CPS security protection, and deep-packet inspection is the primary method for signature-matched mechanisms. This method usually employs regular expression matching (REM) to detect possible threats in the packet payload. State explosion is the critical challenge for REM applications, which originates primarily from features of large character sets with unbounded (closures) or bounded (counting) repetitions. In this work, we propose Offset-FA to handle these repetitions in a uniform mechanism. Offset-FA eliminates state explosion by extracting the repetitions from the nonexplosive string fragments. Then, these fragments are compiled into a fragment-DFA, while a fragment relation table and a reset table are constructed to preserve their connection and offset relationship. To our knowledge, Offset-FA is the first automaton to handle these two kinds of repetitions together with a uniform mechanism. Experiments demonstrate that Offset-FA outperforms state-of-the-art solutions in both space cost and matching speed on the premise of matching correctness, and achieves a comparable matching speed with that of DFA on practical rule sets.

## 1. Introduction

Cyber–physical systems (CPS) are integrations of computational, networking, and physical processes. Embedded computers and networks monitor and control physical processes with feedback loops where physical processes affect computations and vice versa [1]. In the past few decades, we have witnessed exponential growth in the deployment and development of various CPSs [2]. These CPSs affect almost all aspects of our daily lives, such as industrial control systems (ICSs), power grids, medical centers, and transportation systems. On the other hand, cyber–physical systems’ security problems are becoming increasingly serious. The security hazards of cyber–physical systems are spreading from the information domain to the physical domain, and attacks on some critical infrastructures may lead to catastrophic consequences.

For example, Iran’s nuclear power plant was attacked by the Stuxnet worm in 2010, and a vast number of centrifuges (machines that concentrate uranium by spinning at very high speed) were taken out of operation and replaced. Ukraine’s power system was attacked by the BlackEnergy malware in 2015, resulting in power outages in at least three power areas. In 2017, the Triton malware was exploited to attack a petrochemical plant in the Kingdom of Saudi Arabia, causing it to shut down to prevent an explosion [3]. In 2020, SolarWinds, Honda Motors, and the University of Vermont Health Network were attacked with SUNBURST malware, Ekans ransomware, and Ryuk ransomware, respectively [4]. Furthermore, the rapid deployment of the Internet of Things (IoT) and wireless sensor networks (WSNs) has dramatically increased attack access while bringing connectivity.

The security of industrial control systems is generally designed to consider recommendations of the U.S. NIST SP 800-82 Rev.2 and IEC/ANSI/ISA 62443 standards. Intrusion detection systems (IDSs) with deep-packet inspection (DPI) capabilities for filtering industrial control protocols are indispensable elements in implementing the essential security principles, standards, and best practices of IEC 62443 [5]. The employed DPI technology is a form of packet filtering that locates, identifies, classifies, reroutes, or blocks packets with specific data or code payloads that conventional packet filtering that examines only packet headers cannot detect. In deep-packet inspection, attack signatures are usually described as strings. Whether a packet has a threat can be judged by matching the strings with the packet payload byte by byte. However, as signatures are becoming more complicated (e.g., with wildcard characters), using exact strings is no longer an adequate representation. The regular expression (RE) became famous for its flexible and powerful expressive ability, and it is widely used in network applications and devices, such as open-source network intrusion detection systems (NIDSs) Snort [6], Bro [7], Suricata, and the Linux application protocol classifier (L7-filter) [8].

However, it can be challenging for the DPI process to meet requirements on latency and jitter in some real-time communication in industrial automation and control systems where delays and jitters in data delivery can severely impact the effectiveness of the control actions. Therefore, the DPI process should be as fast as possible; the ideal situation is to achieve line-speed processing. For automatic matching in DPI, RE signatures need to be converted into an equivalent finite-state automaton (FSA), where each state represents a different partial matching stage. The matching process is an input-driven state traversal process. During the procedure, any accessed final state denotes recognizing its corresponding patterns. Usually, signatures are first compiled into nondeterministic finite automata (NFA) and then converted into deterministic finite automata (DFA). DFA has only one active state at any time, whereas all NFA states may be activated concurrently in the worst situation. Thus, DFA is more widely used for its superb O(1) time complexity. However, NFA-to-DFA conversion may incur state expansion or even state explosion. In the worst case, the number of DFA states would be $2^{n}$, where *n* is the number of corresponding NFA states. Table 1 shows the worst-case comparisons of NFA and DFA for various strategies. The state expansion of DFA is the biggest threat to line-speed REM because the DFA is too large to be deployed on fast but small on-chip memories.

**Figure 1:**
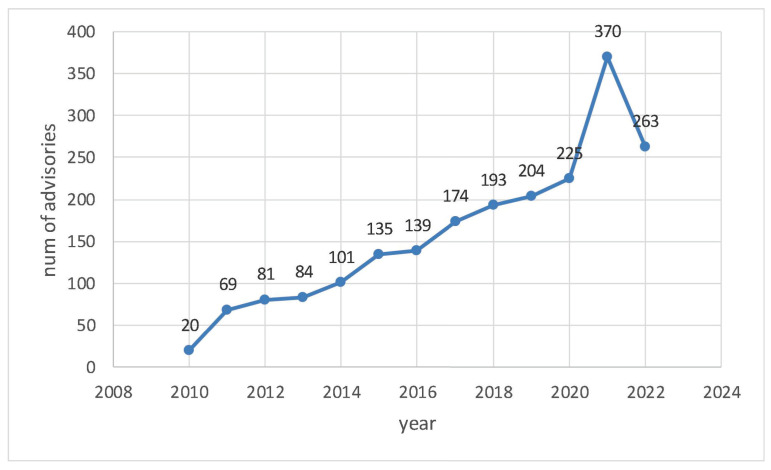
Rapidly increasing ICS-CERT advisories reported by CISA.

State explosion is the origin of all the challenges in the regular expression matching field, and the increasing number and complexity of attack features continue to exacerbate this challenge. We counted the ICS-CERT advisories from the Cybersecurity and Infrastructure Security Agency (CISA). These advisories provide timely information about current security issues, vulnerabilities, and exploits. The results show that advisories are growing rapidly, from 20 in 2010 to 370 in 2021, as shown in Figure 1.

Over the past few decades, related work has searched for solutions to achieve higher performance with moderate platforms. These solutions can be roughly divided into software and hardware solutions. Software solutions exploit automata with compact representations and limited memory bandwidth, and hardware solutions leverage parallel platforms to speed up the overall throughput. Hardware platforms include circuit-centric FPGAs [9,10,11,12] and memory-centric ASICs [13,14], multicore processors [15,16,17], GPUs [18,19,20,21] and TCAMs [22,23,24]. Software solutions and hardware platforms are orthogonal, and we only discuss the software solutions in this paper.

Most studies have focused on compression technologies [25,26,27,28,29,30,31,32] to restrain the DFA expansion problem. Classical compression algorithms such as D${}^{2}$FA [25] and A-DFA [26] were devised to reduce the space cost of DFA with performance guarantees. Many algorithms [26,27,28,29] are based on D${}^{2}$FA. All these algorithms work on the basis of DFA; however, in modern applications, DFA is unavailable due to state explosion. Thus, the above DFA-based compression methods are useless even if they are efficient because all compression algorithms are implemented on the foundation of DFA generation.

Some other works were devoted to designing novel memory-efficient automata to replace DFA. These automata do not need to first generate a DFA, and they can support large and complex rule sets. They are classified as scalable FAs [33], and our Offset-FA also belongs in this category. Typical scalable FAs include multiple DFAs [34,35,36], semidetermined FAs [37,38,39,40,41], decomposed FAs [38,42,43,44,45,46], and various improving proposals. Multiple DFAs prevent state explosion by dividing rule sets into groups, but optimal grouping is an NP-hard problem, and the matching performance degrades linearly as the grouping number rises. Considering that most DFA states have never or rarely been accessed, semidetermined FAs suppress state explosion by controlling the degree of determinism. Among them, hybrid-FA is the most practical automaton. To our knowledge, industrial products such as Cavium’s OCTEON [40] and Broadcom’s XLP7XX/8XX/9XX [41] series processors leverage similar automata for their DPI engines. However, it is not suitable for patterns whose explosive features appear at the beginning of the patterns.

To avoid state explosion, a new direction [38,42,43,44] attaches auxiliary variables to states to record the matching process instead of using states to represent all the possible matching sequences in DFA. These automata are classified as decomposed FAs, and H-FA [38] and XFA [42,43] are the representatives. As closures and counting constraints are represented by labels and counters rather states, the state explosion problem is satisfactorily solved. However, there are still several thorny issues that need further research, which are the direct motivation of our proposal. First, automatic construction is challenging for both H-FA and XFA. Second, false positives (erroneously matched) or false negatives (mismatched) may occur in patterns with unique features [44]. Third, when multiple patterns are compiled together, labels or counters on different transitions may be replicated to each other. As a result, the conditions and instructions on transitions are too complex for efficient processing.

Despite the improvements over the years, few scalable FAs can achieve all the following goals: (i) automatic and fast construction; (ii) semantic equivalence with original signature sets; (iii) small memory footprint; (iv) low memory bandwidth requirement for processing each byte. Here, (i) ensures fast signature update in practice; (ii) ensures the matching correctness; (iii) and (iv) are the central guarantees for the matching performance, but there exists a trade-off between the memory footprint and the memory bandwidth requirements. Almost all the automaton-based solutions focused on providing a preferable trade-off between them. Among all the existing solutions, MFA [45,46] proposed by Norige is similar to our proposal. However, MFA can only handle ‘dot star’ and ‘almost dot star’ features, and other explosive features such as the large character set with counting repetitions remain unsolved. Moreover, even for the ‘dot star’ and ‘almost dot star’ features, the MFA also suffers from potential false positives/negatives as XFA does. The above defects significantly limit the application of MFA in practice.

In this paper, we devise a novel scalable FA called Offset-FA to solve these problems. We attribute the state explosion problem to large character sets with either unbounded (e.g., “.*”) or bounded (e.g., “.{n}”) repetitions. Then, we designed Offset-FA to handle these two kinds of features with a uniform mechanism. In our Offset-FA, the string fragments are extracted from original RE signatures and converted into a fragment-DFA, while a fragment relation table (FRT) and reset table (RT) are constructed to preserve their connection and offset information. Fragment-DFA, and the fragment relation table and reset table constitute Offset-FA. As the origin of explosion (large character sets with repetitions) is eliminated, there is a slight expansion in fragment-DFA. Thus, our Offset-FA could scale to large and complex rule sets. To the best of our knowledge, Offset-FA is the first automaton to handle these two kinds of repetitions together with a uniform mechanism while achieving the above goals.

This article presents the following contributions:

- **Offset-FA:** We propose scalable Offset-FA to handle both unbounded and bounded repetitions with a uniform mechanism (Section 2.1); a formal model (Section 2.3) of Offset-FA is also presented.
- **Generation and matching algorithms:** we provide the automatic generation algorithm (Section 3.1) and matching algorithm (Section 3.2) for Offset-FA while preserving the semantic equivalence.
- **Optimization and implementation details:** practical guidelines for building efficient matching Offset-FA are discussed from both the compilation (Section 4.1) and matching (Section 4.2) aspects. Further, data structures and implementation details are provided (Section 5).
- **Evaluation:** experiments were conducted on practical datasets and compared with state-of-the-art solutions; results demonstrate the features of fast construction, low space cost, and low memory bandwidth requirement in Offset-FA (Section 6).

We proposed a relatively preliminary idea of Offset-FA in a short four-page paper [47] in 2017. Compared with the publication in 2017, the current article conducts systematic and comprehensive exposition of Offset-FA. The new content includes (1) Offset-FA examples for complex features; (2) general processing mechanisms for various features; (3) the formal model definition; (4) the compilation algorithm of Offset-FA; (5) the matching algorithm of Offset-FA; (6) the proof of semantic equivalence between Offset-FA and corresponding regular expressions; (7) optimization for Offset-FA compilation and matching; (8) specific implementation details; (9) comparative experiments and result analysis.

## 2. Offset-FA Proposal

In this section, we first indicate that large character sets with bounded or unbounded repetitions are the leading causes of state explosion. Then, we use several typical examples to illustrate how Offset-FA solves the state explosion problem while keeping semantic equivalence. We also discuss various specific pattern cases for implementation in Offset-FA. Lastly, we provide the formal definition of Offset-FA.

### 2.1. Main Idea

With the ever-increasing scale and complexity of pattern sets in practice, state explosion is an inevitable problem. State explosion originates from the ambiguity of metacharacters in regular expressions because massive states are needed to distinguish all possible input sequences. Generally speaking, there are two kinds of inflation: when compiling a single expression, and when compiling multiple expressions.

Yu [34] analyzed the DFA state number for various pattern features. The large character set with counting repetitions in a single pattern may cause quadratic or even exponential state expansion. The DFA state number of pattern *“ˆA+[ˆ∖n]{4}BC”*, is 22, a quadratic complexity with respect to the length constraint. Quadratic complexity comes from character class *‘[ˆ∖n]’* overlapping with its prefix *‘A+’*. When the first character *‘A’* is matched, input *‘A’*s can match either *‘A+’* or *‘[ˆ∖n]{4}’*. To avoid a false negative, the DFA must record all the situations that would cause quadratic inflation. For another pattern, “.*A.{4}BC”, a large character set with counting repetitions *‘.{4}’* also overlaps with its prefix *‘A’*. Without starting anchor *‘ˆ’*, prefix *‘A’* can appear at any position of the input sequence. Its DFA needs 56 states, which is exponential complexity with respect to the counting constraint.

In practice, a more popular situation for state explosion occurs when compiling multiple patterns into a single DFA, though none of these patterns had state inflation. This explosion is mainly caused by the ‘.*’ feature [34,37], where *‘.’* represents any character, and ‘*’ denotes repeating its previous character from zero to an infinite number of times. Thus, ‘.*’ can represent any input sequence. When compiling multiple patterns together, each ‘.*’ in a pattern generates duplications of states from all other patterns. DFA needs to record the prefixes of these ‘.*’s that were matched, and each combinational situation requires a state. Generally speaking, for *m* patterns with one ‘.*’ in each pattern, we need $2^{m}$ states to record the power set of these prefixes. In fact, a large character class with counting repetitions can cause a similar problem if the counting repetition is large enough.

In practical rule sets, large character sets with bounded or unbounded repetitions are the primary causes of state explosion. To prevent such state explosion, Offset-FA extracts these features from original patterns and compiles the remaining fragments to a DFA, namely, fragment-DFA. Then, a fragment relation table and a reset table are constructed to record the relationships among these fragments. In essence, Offset-FA uses tables rather than a vast number of states to represent these repetitions.

**Figure 2:**
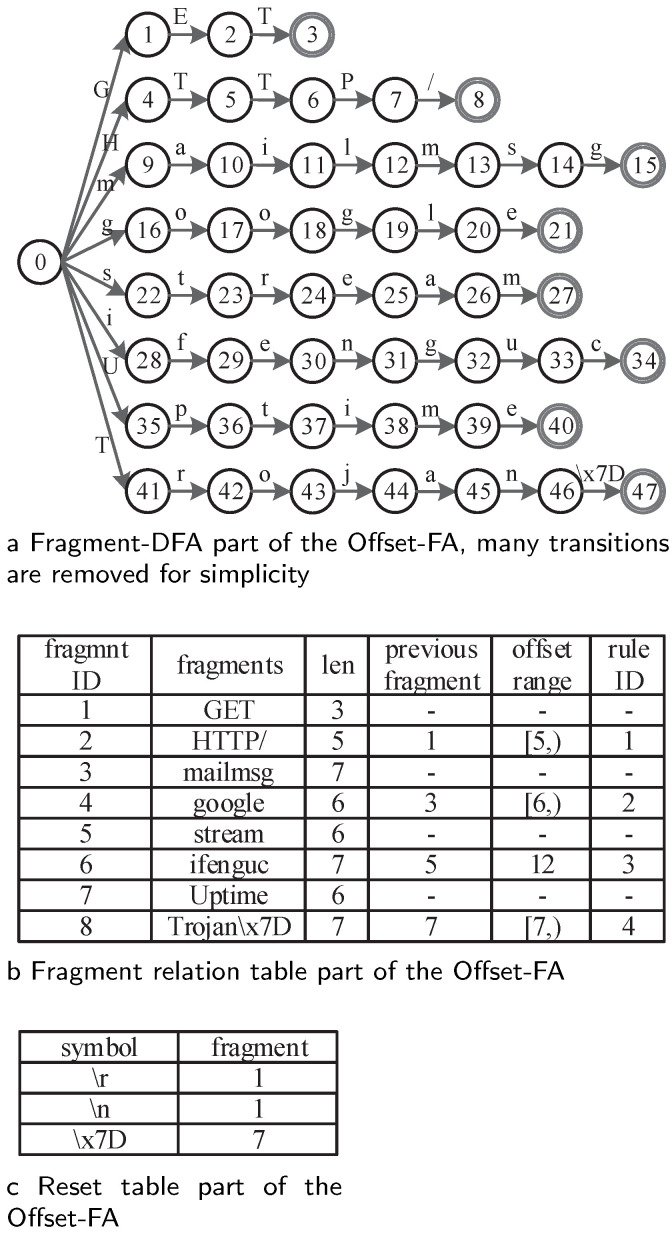
Offset-FA for the rule set GET[ˆ∖r∖n]*HTTP/, mailmsg.*google, *stream.{5}ifenguc*, and Uptime[ˆ∖x7D]*Trojan∖x7D.

To clarify, we used a simple pattern set to explain how Offset-FA handles the state explosion problem while keeping semantic equivalence. Considering rules GET[ˆ∖r∖n]*HTTP/, mailmsg.*google, *stream.{5}ifenguc*, and Uptime[ˆ∖x7D]*Trojan∖x7D as the existing large character sets with unbounded or bounded repetitions, the corresponding DFA requires 996 states to describe these rules. In total, 48 states are enough in Offset-FA, as shown in Figure 2.

Offset-FA comprises three parts: fragment-DFA, fragment relation table, and reset table. The extracted fragments are compiled to fragment-DFA, which is a DFA for fragment matching. After removing the large character sets with repetitions, the fragments are mainly simple strings. Thus, there is slight state expansion in fragment-DFA. Large character sets with repetitions are compiled to the fragment relation table (FRT) and the reset table (RT) for checking and keeping semantic equivalence. The fragment relation table records the length, previous fragment ID, offset range requirement, and corresponding rule ID for each fragment. Here, the matching of a fragment is checked as valid if its matched position minus the matched position of its previous fragment fulfils the offset range. In addition, we need a reset table to handle *‘[ˆ∖n]’*-like large character set, which may invalidate the matching of its previous fragment.

Next, we used several input sequences to explain how Offset-FA works for matching. For each input sequence, the matching engine maintains a matching process table (MPT) to record the current matching status, recording the fragments and the matched positions that were matched. The MPT is checked and updated during the matching process.

**Figure 3:**
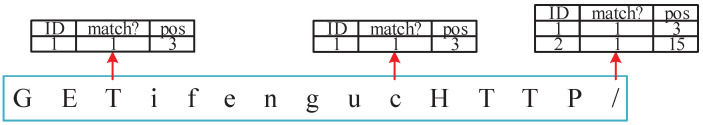
Matching process for the input *GETifengucHTTP/*.

For input *GETifengucHTTP/*, the matching engine creates an empty MPT and starts from the beginning of the input. After reading *GET*, State 3 is active in fragment-DFA, and fragment *“GET”* is matched. Then, the matching engine needs to inquire the fragment relation table to obtain the information of the fragment *“GET”*. As no previous fragment is required before *“GET”*, the matching of *“GET”* is confirmed, and the matching information is updated to the MPT. Then, for the following *ifenguc*, State 34 is active, and fragment *“ifenguc”* is matched. However, from FRT, we know that fragment *“ifenguc”* relies on the matching of its previous fragment *“stream”*. As fragment *“stream”* has not yet appeared in the matching process table, the matching of fragment *“ifenguc”* is a false positive and is not updated to the MPT. After reading the remaining sequence, *HTTP/*, State 8 is active, and we need to inquire about information on Fragment 2. According to FRT, Fragment 2 relies on Fragment 1. As Fragment 1 was recorded in the MPT, we needed to further check whether the offset requirement was fulfilled. The matched positions of Fragments 1 and 2 were 3 and 15, respectively, and their position offset was 12. This fulfils the offset range [5); thus, the matching of Fragment 2 is valid, and its corresponding rule ID 1 is reported. Figure 3 shows how the MPT is updated during matching.

**Figure 4:**
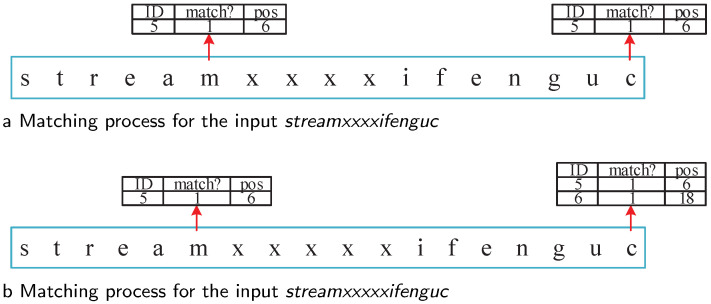
Matching process for inputs *streamxxxxifenguc* and *streamxxxxxifenguc*.

Similarly, for input sequence *streamxxxxifenguc*, Fragment 5 is marked as matched at Position 6 after the processing *stream*. When the last character is processed, State 34 is active, and Fragment 6 is matched at Position 17. From the FRT, we know that Fragment 6 relied on Fragment 5, but their position offset was 11, not equal to the offset requirement 12. Thus, the matching of Fragment 6 is a false positive. For input *streamxxxxxifenguc*, Fragments 5 and 6 are matched at Positions 6 and 18, respectively. Their position offset was equal to the offset range 12; thus, Fragment 6 was matched, and its corresponding rule ID 3 was reported. The matching processes for these two sequences are shown in Figure 4.

**Figure 5:**
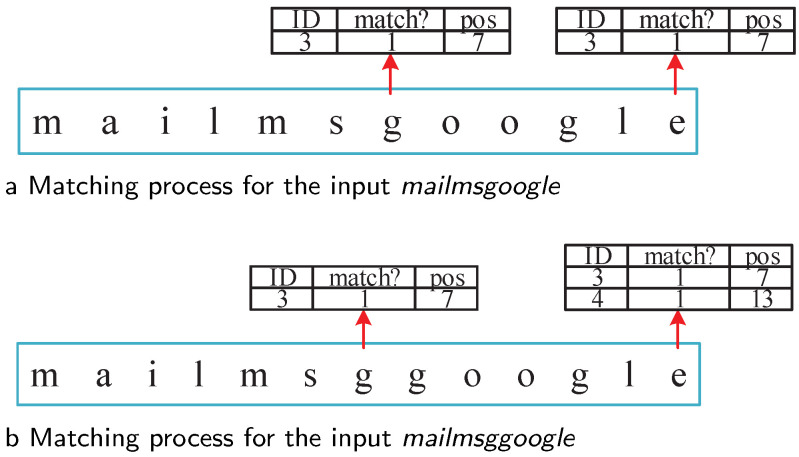
Matching process for inputs *mailmsgoogle* and *mailmsggoogle*.

In RE pattern “mailmsg.*google”, fragment *‘mailmsg’* overlapped with fragment *‘google’* on character*‘g’*. This is a typical situation that would cause false positives in traditional methods [44], but Offset-FA could handle it correctly. For input *mailmsgoogle*, Fragments 3 and 4 were matched at Positions 7 and 12, respectively. The position offset was 5, not belonging to offset range [6,), thus the matching of fragment 4 is a false positive. For input *mailmsggoogle*, Fragments 3 and 4 are matched at Positions 7 and 13. Ttheir position offset 6 fulfills the range [6,). Thus the corresponding rule ID 2 is reported. For this kind of RE pattern, no false positives or false negatives occur in Offset-FA. Figure 5 displays the corresponding matching process.

For large character set ‘.’, we only needed to consider the offset relationships among the corresponding fragments. However, for the *‘[ˆc]’*-like character set, we had to also consider the situations in which excluded character *c* appears in the input sequence. For this kind of situation, another reset position field is required in the matching process table. The following examples show how these situations may cause potential false positives or false negatives and how Offset-FA handles these situations with the aid of the reset table.

**Figure 6:**
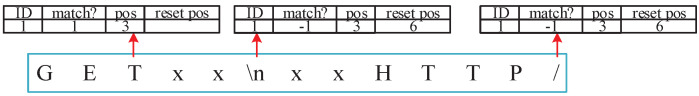
Matching process for input *GETxx∖nxxHTTP/*.

For input *GETxx∖nxxHTTP/*, Fragment 1 is matched at Position 3, and this matching status is updated to the MPT. Then, the MPT has no change until the matching engine reads character *∖n* at Position 6. The reset table shows that character *∖n* would cause the reset operation for Fragment 1. Thus, the matching information of Fragment 1 is reset in MPT, as shown in Figure 6. Here, −1 denotes that the fragment was matched before but reset later. The reset position value is also updated to the MPT and is used to avoid false positives and false negatives. When the last character was processed in fragment-DFA, State 8 was active. Then, we needed to check the corresponding requirements for Fragment 2. Its previous fragment was reset at Position 6. From the matched position (13) and length (5) of Fragment 2, we know that the reset position was actually before Fragment 2. This means that the reset operation was valid, and the matching of Fragment 2 was a false positive and not recorded.

**Figure 7:**
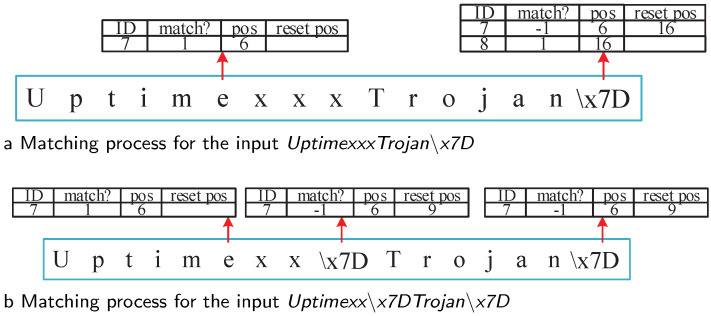
Matching process for inputs *UptimexxxTrojan∖x7D* and *Uptimexx∖x7DTrojan∖x7D*.

The situation becomes more complex if the excluded character appears in the following fragment in the RE pattern [44]. For example, in pattern “Uptime[ˆ∖x7D]*Trojan∖x7D”, excluded character *‘∖x7D’* appears in fragment *‘Trojan∖x7D’*. The matching engine must be able to distinguish between different reset situations. For input *UptimexxxTrojan∖x7D*, Fragment 7 is first matched at Position 6. Then, after processing *xxxTrojan∖x7D*, State 47 was active in Fragment-DFA. For the last character, *∖x7D*, the engine reset the corresponding Fragment 7, and the reset position was set as 16. Then, for matched Fragment 8, the engine checked that Fragment 7 is reset at Position 16. According to the matched position (16) and length (7) of Fragment 8, the reset position was inside Fragment 8, which means that the reset was caused by the matching of Fragment 8. Thus, this reset is an invalid operation, and the matched information of Fragment 8 was updated to the MPT. Further, corresponding rule ID 4 was reported. The matching process is illustrated in Figure 7a.

For another input, *Uptimexx∖x7DTrojan∖x7D*, excluded character *∖x7D* appeared twice. To avoid false positives, the matching engine should only execute and record the first reset operation, as shown in Figure 7b. Similarly, Fragment 7 was set as matched at Position 6 and reset at Position 9. Then, when processing the last character, *∖x7D*, Fragment 8 was matched. As the character *∖x7D* appeared in the reset table, the matching engine had to also check the corresponding Fragment 7 in the matching process table. Fragment 7 was reset before, and the engine only recorded the first reset position. Thus, there was no change in the MPT. According to similar procedures, the reset operation of Fragment 7 is before Fragment 8. Thus, it was a valid reset operation; the matching of Fragment 8 was invalid and not recorded in the MPT.

These RE patterns and input examples are typical situations for a large character set with unbounded or bounded repetitions. Next, we discuss various general cases about large character sets with repetitions.

### 2.2. Solutions for Various General Patterns

The main idea of Offset-FA is to divide the patterns at the features of large character sets with repetitions (*LCSR*) and reassemble them with the fragment relation table and reset table. An RE pattern can be expressed as in (1). Here, *LCSR*s can be regarded as the split points, and *frag*s denote the substrings (or fragments) except *LCSR*s.

(1) $$frag_{1}LCSR_{1}frag_{2}LCSR_{2}\cdots LCSR_{k-1}frag_{k}$$

As long as these fragments can be matched and confirmed sequentially, the RE pattern is checked when the last fragment is matched and confirmed. Hence, the matching problem can be reduced to matching a series of $frag_{i}LCSR_{i}frag_{i+1}$.

Table 2 displays various *LCSR* features and corresponding configurations in the fragment relation table. For each $frag_{i}LCSR_{i}frag_{i+1}$, the fragment length field, previous fragment field, and rule ID field for each fragment are clear. Thus, we only discuss the offset range field for each $frag_{i+1}$, as listed in the second column. Here, $Len_{frag}$ denotes the length of the fragment.

In this table, the upper part concludes unbounded *LCSR* features, especially typical ‘.*’ and ‘[ˆc]*’ features. These two kinds of features are the most common in practice. For ‘.*’ and ‘.+’ features, $frag_{i}$ and $frag_{i+1}$ being matched sequentially was not sufficient to demonstrate $frag_{i}LCSR_{i}frag_{i+1}$ was matched. The matching engine must rely on the offset information to omit false positives when $frag_{i}$ overlaps $frag_{i+1}$ (e.g., pattern “ab.*bc”). For ‘[ˆc]*’ and ‘[ˆc]+’, except for the above false positives, we also need to consider situations in which the excluded character c appears in $frag_{i+1}$ (e.g., pattern “ab[ˆc]*cd”), which may cause false positives or false negatives. The matching engine cannot handle such complex features with only matched positions. With the assistance of reset information from the reset table and matching process table, the engine can accurately judge whether the reset of $frag_{i}$ and the matching of $frag_{i+1}$ are valid.

The lower part of Table 2 describes bounded *LCSR* features, among which ‘.{n}’ and ‘[ˆc]{n}’ are the most common in practice. It is relatively easy to handle ‘.{n}’ features because the engine only needs to compute the offset distance between the matched positions of $frag_{i+1}$ and $frag_{i}$. Other features, such as ‘.{m,n}’, ‘.{,m}’ and ‘.{n,}’, are similar to ‘.{n}’. For ‘[ˆc]{n}’ features, it is also necessary to consider false positives/negatives caused by reset operations.

Here, we only provide basic configurations for various general *LCSR* features. The detailed generation algorithm for Offset-FA and the matching algorithm are discussed in Section 3. Next, we first introduce the formal model of Offset-FA.

### 2.3. Formal Model

We formally define an Offset-FA as follows.

**Definition** **1.** *Offset-FA is a 7-tuple (*Q*,*T*,Σ,δ,φ,($q_{0}$,$t_{0}$),*F*), where*

- Q* is the set of states in Fragment-DFA;*
- T* is the set of values in the matching process table;*
- *Σ is the set of input characters;*
- *δ:* Q×Σ→Q*represents the standard input-driven state transition function for Fragment-DFA;*
- *φ: Σ×*Q×T→T*is the update function for the matching process table. Given the input character, current state, and T, it updates the status of the MPT;*
- *($q_{0}$,$t_{0}$), where $q_{0}$∈*Q*and $t_{0}$∈*T*are initial configurations;*
- F∈Q×T*is the set of accepting configurations in Offset-FA.*

As we explained in Section 2.1, the original RE patterns are divided into fragments, and Fragment-DFA is the basic data structure for fragment matching. Fragment-DFA is similar to the standard 5-tuple DFA except that the accepted states in Fragment-DFA denote the matching of fragments rather than patterns. To match the original RE patterns, it must rely on the matching process table to confirm the sequential matching of the fragments. Hence, compared with the standard 5-tuple DFA, Offset-FA introduces the domain of *T* to describe the MPT’s status and a function φ to update it. δ is an input-driven state transition function of Fragment-DFA. Each time a fragment is recognized in Fragment-DFA, the matching engine invokes the φ function to update the MPT and confirm the matching of the fragment. Once a tail fragment (the last fragment of a pattern) is confirmed, the engine reports its corresponding pattern ID.

## 3. Generation and Matching Algorithm for the Offset-FA

In the previous section, we demonstrated that Offset-FA can efficiently handle the state explosion caused by *LCSR* features while keeping semantic equivalence. In this section, we provide the algorithm to compile RE patterns to Offset-FA and the matching algorithm in Offset-FA. Further, we also prove the semantic equivalence between Offset-FA and the original RE pattern set.

### 3.1. Offset-FA Generation Algorithm

The generation algorithm consists of three phases. First, the RE patterns are annotated according to their *LCSR* features. Second, on the basis of the annotation, the RE patterns are split into fragments, and the fragment relation table and reset table are constructed. Lastly, the fragments are compiled to Fragment-DFA.

The pseudocode of the generation algorithm is shown in Algorithm 1. Steps 3 to 5 recognize the *LCSR* features in each RE pattern. There exists a trade-off between the space cost and matching efficiency in Offset-FA compilation. This depends on how *LCSR* features are defined. For small character sets (e.g., [0–9]*) or large character sets with small bounded repetitions (e.g., [ˆn]{3}), it is better to leave them in the fragments rather than regard them as *LCSR* features. If these features are regarded as fragments and compiled to Fragment-DFA, they cause a slight state expansion. However, if they are regarded as *LCSR* features, and compiled to FRT and RT, they may incur frequent update operations during matching that would significantly affect matching performance. We discuss this issue more in Section 4. Steps 6 to 9 mark the recognized *LCSR* features in each pattern, and all *LCSR* features are enclosed between symbols ⊳ and ⊲.

**Algorithm 1** Offset-FA generation algorithm
**Require:** regular expression pattern set $S_{RE}$;**Ensure:** corresponding Offset-FA, including fragment-DFA, a fragment relation table (FRT), and a reset table (RT);1:$Q_{frag}$ = ∅, FRT = ∅, RT = ∅;2:
*//Phase 1: annotating the regular expressions*
3:**for** each $p\in S_{RE}$ **do**4:      RE.recognize_LCSRs(p);5:
**end for**
6:**for** each LCSR∈p.LCSRs **do**7:      RE.mark (LCSR.head,⊳);8:      RE.mark (LCSR.tail,⊲);9:
**end for**
10:
*//Phase 2: split the patterns into fragments, and generate the fragment relation table and reset table*
11:**for** each $p\in S_{RE}$ **do**12:      prev_frag = null; prev_LCSR = null;13:      cur_frag = RE.read_next_frag(p);14:      **repeat**15:            $Q_{frag}$.push(cur_frag);16:            FRT.set(prev_frag,prev_LCSR,cur_frag);17:            **if** character set of prev_LCSR is not ‘.’ **then**18:                 RT.set(prev_frag, prev_LCSR);19:            **end if**20:            prev_frag = cur_frag;21:            prev_LCSR = RE.read_next_LCSR(p);22:            cur_frag = RE.read_next_frag(p);23:        **until** cur_frag == null or prev_LCSR == null24:
**end for**
25:
*//phase 3: compile the fragments to fragment-DFA*
26:fragment-NFA = re_to_nfa($Q_{frag}$);27:fragment-DFA = nfa_to_dfa(fragment-NFA);28:dfa_minimize (fragment-DFA);29:**return** Offset-FA (fragment-DFA, FRT, RT);


Steps 11 to 24 correspond to Phase 2. The patterns are parsed and split into fragments according to the marks in Phase 1, and the corresponding fragment information is added to the fragment relation table and fragment reset table. For each pattern, the fragments are sequentially pushed to queue $Q_{frag}$ (Step 15). According to the previous fragment and the previous *LCSR* feature, Step 16 computes the offset range field for the current fragment. The corresponding rule ID is also attached if the current fragment is the last fragment. If the character set of the *LCSR* feature is not ‘.’, the reset information for the excluded characters should be added to the reset table (Steps 17 to 19). Taking the pattern “Uptime[ˆ∖x7D]*Trojan∖x7D” as an example, excluded character ‘∖x7D’ should be added to the reset table to avoid possible false positives during matching. Lastly, Steps 26 to 28, which are same as the standard DFA generation algorithm, are used to generate Fragment-DFA.

The above generation algorithm runs well in general situations. However, some exceptional cases need more consideration. In fact, there is a suppressed premise for correctly matching in Offset-FA. Namely, all split fragments should be distinct. Otherwise, false positives may occur for some inputs. For example, two patterns, “ab.*cd.*ef” and “mn.*cd.*gh”, are split according to the above generation algorithm into the following fragments: *“ab”*, *“cd”*, *“ef”*, *“mn”*, *“gh”*. Obviously, two patterns share the same fragment, *“cd”*. For input *abcdgh*, substring *abcd* sequentially activates the matching status of fragments *“ab”* and *“cd”*. Then, the following substring, *gh*, matches fragment *“gh”*. As its previous fragment, *“cd”* was marked as matched, the offset range was fulfilled, and fragment *“gh”* was marked as matched. Then, corresponding pattern “mn.*cd.*gh” was reported as matched, and a false positive occurred.

The false positive originated from two patterns shaing the same fragment, *“cd”*. Fragment *“gh”* was judged as matched only according to the matching status of its previous fragment, *“cd”*. However, the matching status of *“cd”* was not the partial matchomg of pattern “mn.*cd.*gh”. It was actually the partial matching of the other pattern, “ab.*cd.*ef”. Similar false positives may appear if a pattern contains two or more identical fragments.

To avoid the above false positives, when a fragment is matched, the matching engine needs to query the matching status of its previous multihop neighbor fragments rather than just one hop neighbor fragment to confirm the matching of the current fragment. For the above example, when fragment *“gh”* was matched, the matching engine had to check the matching status of both fragments *“cd”* and *“mn”*. This extra query would require the fragment relation table to record more neighbor information for some fragments, which would harm the regularity of the FRT data structure. A performance penalty is inevitable when this kind of fragments is matched frequently.

**Figure 8:**
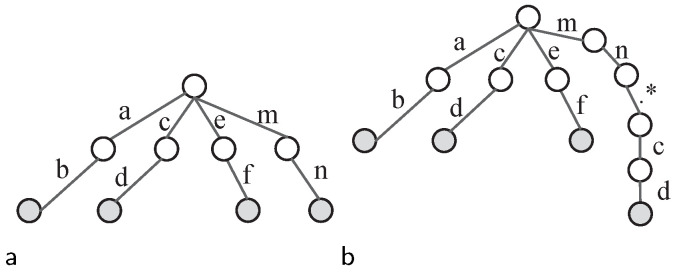
Trie tree construction process when inserting pattern “mn.*cd.*gh”. (**a**) After inserting fragment *“mn”*; (**b**) after inserting fragment *“cd”*.

Considering that the above situations are not very common in practice, we employed another method to avoid possible false positives. During the split procedures of Offset-FA generation, a trie tree is constructed to record the achieved fragments through splitting. Its fragments are sequentially added to the trie tree for a new pattern. If the same fragment is found in the trie tree when inserting a fragment, this pattern has the same fragment with another previous pattern. Rather than being regarded as an individual fragment, this fragment is appended to the tail of its previous fragment along with the LCSR features between them. This newly combined fragment is regarded as a fragment in Offset-FA. Figure 8 explains the construction process of the trie tree when inserting the fragments in pattern “mn.*cd.*gh”. Fragment *“cd”* was combined with its previous fragment in Figure 8b to avoid the shared fragment among patterns.

This method introduces some state expansion, but the expansion is very limited because it is not a typical case in practice. In fact, a more common case is that multiple patterns share the same fragment located at the head of each pattern. For example, in the tested spyware-put rule set from the Snort NIDS [6], 174 patterns shared the same fragment, *“ˆHost∖x3A”*, 77 patterns shared the same fragment, *“ˆUser-Agent∖x3A”*, and 14 patterns shared the same fragment, *“ˆX-Mailer∖x3A”*. Obviously, all shared fragments were located at the head of their corresponding patterns. In such situations, as the shared head fragments had no previous fragment that needed further confirmation, we could quickly figure out that they would not cause any false positives during matching. Thus, we did not need to handle the shared head fragments as in Figure 8.

### 3.2. Matching Algorithm

The matching procedures in Offset-FA are explained in Algorithm 2. This is mainly composed of two parts: the input-driven state transition in Fragment-DFA and the update of the matching process table. The former is similar to DFA matching; given the current state and input character, Fragment-DFA returns the new current state (Step 4). Further, if the character c appears in the reset table, the matching engine should also update the reset information for its corresponding fragments in the MPT (Steps 5 to 9). Only the corresponding matched fragments should be reset, and those fragments that were reset should remain unchanged. The situation becomes complex if the new current state is an accepting state (Step 10) that invokes function φ to update the MPT (Step 11). Then, if the updated MPT satisfies the matching conditions, the corresponding rule ID is reported as matched.
**Algorithm 2** Matching algorithm for the Offset-FA**Require:** Offset-FA (Fragment-DFA, fragment relation table (FRT), reset table (RT)), a packet payload;**Ensure:** the matched rule ID(s);1:cur_state = $q_{0}$; MPT = ∅; matched_rules = ∅;2:**for**i=0 to payload.len **do**3:      c = payload.getchar(i);4:      cur_state = δ (cur_state,c); *//state transition*;5:      **if** c appears in reset table **then**6:            **for** each fragid ∈ RT.get_fragid(c) **do**7:                 MPT.reset (fragid,*i*); *//reset*;8:            **end for**9:        **end if**10:      **if** fragment-DFA.accepted (cur_state) **then**11:            **MPT = φ (c,cur_state,MPT)**; *//update*;12:            **if** (cur_state,MPT)∈*F* **then**13:                 rule_id = FRT.get_ruleid (fragment-DFA.14:get_fragid (cur_state));15:                 matched_rules.add (rule_id);16:            **end if**17:      **end if**18:**end for**19:**return** matched_rules;20: 21:*//the MPT update function*;22:**function**φ (c,cur_state,MPT)23:cur_frag = fragment-DFA.getid (cur_state);24:prev_frag = FRT.get_previd (cur_frag);25:**if** prev_frag!=null **then**26:      **if** MPT.is_matched (prev_frag) == 1 **then**27:            **if** *i*-prev_frag.match_pos∈28:MPT.get_range (cur_frag) **then**29:                 MPT.update(cur_frag,1,i,0);30:            **end if**31:      **else if** MPT.is_matched (prev_frag) == −1 **then**32:            **if** perv_frag.match_pos >*i*-FRT.get_len (cur_frag) & *i*-prev_frag.match_pos∈33:MPT.get_range(cur_frag) **then**34:                 MPT.update (cur_frag,1,i,0);35:            **end if**36:      **end if**37:**end if**38:**return**MPT;

The primary function of MPT is to confirm the validity of the current matched fragment. To omit various false positives and false negatives, the MPT update function (Steps 22 to 38) was well-designed but is a little complex. If the current fragment is not the first for a pattern, the matching status of its previous fragment and the offset between them need to be verified.

**Figure 9:**
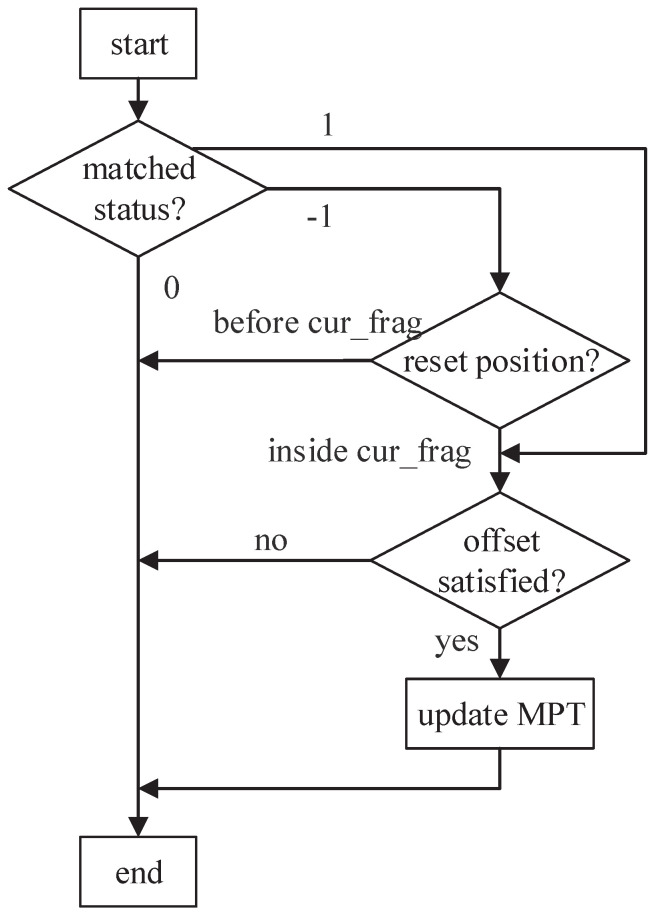
Specific procedures to update the MPT.

There exist three matching statuses (1,−1,0) for the previous fragment in the MPT. Figure 9 clarifies the specific procedures for these cases. If the matching status is 1, the engine only needs to check whether the offset of their matched position satisfies the corresponding offset range. If it is met, the matching of the current fragment is confirmed and updated to the MPT (Steps 26 to 30). Otherwise, the matching of the current fragment is invalid, and the MPT remains unchanged.

If the matching status is −1, the previous fragment is matched before but reset later. Obviously, the reset position only has two situations: located before the current fragment (and after the matched position of the previous fragment) or located inside the current fragment. In the former case, the previous fragment is reset by the *LCSR* feature between these two fragments (e.g., Figure 6). It is a valid reset operation; thus, the current fragment is regarded to be unmatched. In the latter case, the previous fragment is reset by the characters from the current fragment (e.g., Figure 7a). Hence, it is an invalid reset operation. Further, if the offset condition is also satisfied, the current fragment can be confirmed as matched (Steps 32 to 34).

If the matching status is 0, which means that the previous fragment was never matched, the matching of the current fragment is also invalid.

### 3.3. Demonstration of Semantic Equivalence

In the above subsection, we provide the matching algorithm for the Offset-FA, and an RE pattern is reported once the matching of its last fragment is confirmed. Although it is obvious to find the semantic equivalence between the RE patterns and Offset-FA, we still need to provide theoretical proof. The demonstration is proven from two aspects: (1) necessity, with the confirmed matching of a last fragment in Offset-FA proving the matching of its corresponding RE pattern; (2) sufficiency, with an input sequence matching an RE pattern proving that the last fragment of the pattern can also be matched and confirmed in the corresponding Offset-FA.

Proof of necessity.

**Proof.**  In Algorithm 2, the confirmed matching of a fragment means its previous fragment is matched, and the *LCSR* feature between them is also satisfied. From the confirmed matching of the last fragment (supposing *frag*${}_{k}$), the previous fragment *frag*${}_{k-1}$ is matched, and the *LCSR* feature *LCSR*${}_{k-1}$ is also satisfied, namely, the combined larger fragment *frag*${}_{k-1}$*LCSR*${}_{k-1}$*frag*${}_{k}$ is also matched and confirmed. Further, with the confirmed matching of *frag*${}_{k-1}$, we can similarly prove the confirmed matching of *frag*${}_{k-2}$ and *frag*${}_{k-2}$*LCSR*${}_{k-2}$*frag*${}_{k-1}$. Lastly, for all i∈ [2,*k*], we obtained the confirmed matching of *frag*${}_{i-1}$ and *frag*${}_{i-1}$*LCSR*${}_{i-1}$*frag*${}_{i}$.

In turn, given the confirmed matching of *frag*${}_{1}$*LCSR*${}_{1}$*frag*${}_{2}$, we regarded it as the new fragment, *frag*${}_{12}$. *frag*${}_{3}$ is matched, and the *LCSR* feature *LCSR*${}_{2}$ between fragments *frag*${}_{12}$ and *frag*${}_{3}$ is also satisfied. Hence, combined fragment *frag*${}_{12}$*LCSR*${}_{2}$*frag*${}_{3}$ (named *frag*${}_{123}$) is also matched. Similarly, we can subsequently obtain the confirmed matching of *frag*${}_{123}$*LCSR*${}_{3}$*frag*${}_{4}$, *frag*${}_{1234}$*LCSR*${}_{4}$*frag*${}_{5}$, and finally the *frag*${}_{12...k-1}$*LCSR*${}_{k-1}$*frag*${}_{k}$. The last confirmed matching fragment *frag*${}_{12...k-1}$*LCSR*${}_{k-1}$*frag*${}_{k}$ is the original RE pattern. □

Proof of sufficiency.

**Proof.**  The precondition is an RE pattern (supposing *frag*${}_{1}$*LCSR*${}_{1}$*frag*${}_{2}$⋯*LCSR*${}_{k-1}$*frag*${}_{k}$) and an input sequence (supposing $c_{1}c_{2}$⋯$c_{n}$) that matches this pattern. We split the pattern into *frag*${}_{1}$|*LCSR*${}_{1}$|*frag*${}_{2}$|⋯|*LCSR*${}_{k-1}$|*frag*${}_{k}$; each *frag*${}_{i}$ or *LCSR*${}_{i}$ is also a valid subpattern. As the sequence matches the original pattern, its subpatterns must be matched by the subsequences one by one. Here, we supposed that *frag*${}_{1}$ was matched by $c_{1}c_{2}$⋯$c_{m_{1}}$, *LCSR*${}_{1}$ was matched by $c_{m_{1}+1}c_{m_{1}+2}$⋯$c_{m_{2}}$, and *frag*${}_{2}$ was matched by $c_{m_{2}+1}c_{m_{2}+2}$⋯$c_{m_{3}}$. Similarly, *frag*${}_{k-1}$ was matched by $c_{m_{2k-4}+1}c_{m_{2k-4}+2}$⋯$c_{m_{2k-3}}$, *LCSR*${}_{k-1}$ was matched by $c_{m_{2k-3}+1}c_{m_{2k-3}+2}$⋯$c_{m_{2k-2}}$, and *frag*${}_{k}$ was matched by $c_{m_{2k-2}+1}c_{m_{2k-2}+2}$⋯$c_{n}$. Further, according to Algorithm 2, the matching of *frag*${}_{2}$ was confirmed because its previous *frag*${}_{1}$ was matched, and *LCSR*${}_{1}$ was satisfied. Similarly, the matching of *frag*${}_{3}$ was confirmed because its *frag*${}_{2}$ was matched and *LCSR*${}_{2}$ was satisfied. Lastly, we could ensure the matching of *frag*${}_{4}$, *frag*${}_{5}$⋯, *frag*${}_{k-1}$ and *frag*${}_{k}$. □

## 4. Optimizations

In the introduction, we indicated the main goals in REM: automatic construction, semantic equivalence, small memory footprint, and low-memory bandwidth requirements. The generation algorithm and demonstrations in Section 3.3 solved the two former issues. With the separation of *LCSR* features, Offset-FA is expected to perform very well in terms of space cost. Meanwhile, it should also provide the low-memory bandwidth requirement for high-speed matching.

There exists a trade-off between the space cost and matching speed in all automaton-based REMs. NFA and DFA are the two extremes among these automata. The ideal automaton should have space cost as low as that of NFA and matching speed as fast as that of DFA, but this is impractical. As Offset-FA performs well in space cost, in this section, we provide some general guidelines to improve its matching performance.

From Algorithm 2, we know that the matching of Offset-FA is composed of two parts, state transition in Fragment-DFA and the update of the MPT. The state transition in Fragment-DFA is similar to the matching in DFA and has a fixed O(1) time complexity. Thus we focus on the second part, improving the update speed for MPT. Next, we optimize the MPT update efficiency from two aspects: Offset-FA generation in Section 4.1 and Offset-FA matching in Section 4.2.

### 4.1. Optimization for Building Efficient Offset-FA

During the matching process, the MPT needs to be updated on the premises of either the input character incurring the reset operation for some fragments or a fragment being matched in Fragment-DFA. For a given input sequence, the input characters are unalterable. Hence, we mainly discuss the second premise. The update operation involves checking, computing, and set operations. Clearly, it is more complex than state transition in fragment-DFA. This slows down the matching speed if some fragments are frequently matched.

To alleviate this situation, the fragments should be matched as little as possible. This could be achieved by splitting the patterns into longer fragments because longer fragments have lower a probability of being matched. However, longer fragments usually incur more state expansion when compiled into Fragment-DFA. Next, we provide several general guidelines to build better fragments with lower matching probability while incurring slight state expansion.

**(1) Avoid short fragments.** Short fragments may be frequently matched, and each matched instance involves an update operation for the MPT. In the original algorithm, the patterns are split according to the border of each *LCSR* feature. However, if executed strictly, we may obtain many tiny fragments. For example, the original algorithm splits pattern “abcd.*e.*fghik” into fragments ‘abcd’, ‘e’, and ‘fghik’. If the input characters are uniformly distributed, fragment ‘e’ is matched at any position with the high probability of $\frac{1}{256}$. Further, if the length of input sequence is *n*, the matching number of fragment ‘e’ is $\frac{n}{256}$. A better solution is to divide the pattern into ‘abcd.*e’ and ‘fghik’. Although this aggravates the state expansion to some extent, it is still worth it, as the matching probability of ‘abcd.*e’ is highly reduced to about $\frac{n}{256^{5}}$. Hence, we obtain the first guideline: if a fragment is too short (the length is less than 3), then it should be combined with its neighboring fragment rather than be compiled as an independent fragment.

**(2) Strict definition for a large character set.** In practical rule sets, we can find various kinds of character sets such as ‘.’, ‘[ˆ∖n]’, ‘[ˆ∖r∖n]’, ‘[0-9A-z]’, ‘∖d’, and ‘∖s’. They cover the different ranges of the ASCII character code from ‘∖x00’ to ‘∖xff’. However, it is not necessary to regard all these character sets as *LCSR* features, especially when the range is small because a small range character set with repetition incurs very limited semantic overlapping with other patterns. For example, feature ‘[0-9]*’ only partly overlaps the patterns that start with digits, and this incurs a slight state expansion when compiled into DFA. Another motivation is that the excluded characters from the character set would incur reset operation for the MPT. Large character sets have fewer excluded characters than a small character set does, thus incurring fewer reset operations during matching. Hence, the second guideline is that a character set is not regarded as a component of *LCSR* features unless it covers more than 250 symbols.

**(3) Strict limit for bounded repetitions.** For the bounded *LCSR* feature, the repetition of a large character set is limited to a value or a range. If the number of required repetitions is small enough, it does not overlap with other fragments too much. It is still unnecessary to regard these repetitions as *LCSR* features. For example, in the “ab.{3}cd” pattern, unbounded *LCSR* feature ‘.{3}’ brought very limited state expansion when compiled to DFA. Hence, this pattern should be compiled as an integrated fragment rather than split into fragments of ‘ab’ and ‘cd’, which are easily matched. Our third guideline is that the bounded repetition should not be compiled as an *LCSR* feature if its repetition number is less than 4.

According to the above guidelines, we could reasonably generate Fragment-DFA without much state expansion. Further, as Fragment-DFA is a standard DFA, all existing DFA compression algorithms [25,26,27,28] can be employed to reduce the space cost of Fragment-DFA.

### 4.2. Optimizations for Matching Process

Algorithm 2 provides the standard matching process in Offset-FA. The MPT update function is invoked when a fragment is matching, and it involves a series of judgments and operations. However, further analysis shows that the reset validation test and offset condition test are unnecessary for most situations, especially for unbounded LCSR features. In fact, the purpose of the reset validation and offset condition tests is to avoid possible false positives or negatives. Still, if the premises for the false positives/negatives do not exist, these tests would be redundant.

First, for $frag_{1}.*frag_{2}$ features, if the current matched fragment $frag_{2}$ has no overlap with its previous fragment $frag_{1}$, then there is no need to check the corresponding offset condition. For $frag_{1}.*frag_{2}$ cases, the primary purpose of the offset condition test is to eliminate the false positives caused by the overlaps between $frag_{1}$ and $frag_{2}$. If $frag_{1}$ is matched before $frag_{2}$ and there is no overlap between them, the matching path of fragment $frag_{2}$ is not started until $frag_{1}$ is matched. Thus, $frag_{2}$ can only be matched after $frag_{1}$, and this fulfils original feature $frag_{1}.*frag_{2}$, and no false positive occurs. Hence, if a fragment does not overlap with its previous fragment, and the LCSR feature between them is ‘.*’, there is no need to check the offset condition further.

**Figure 10:**
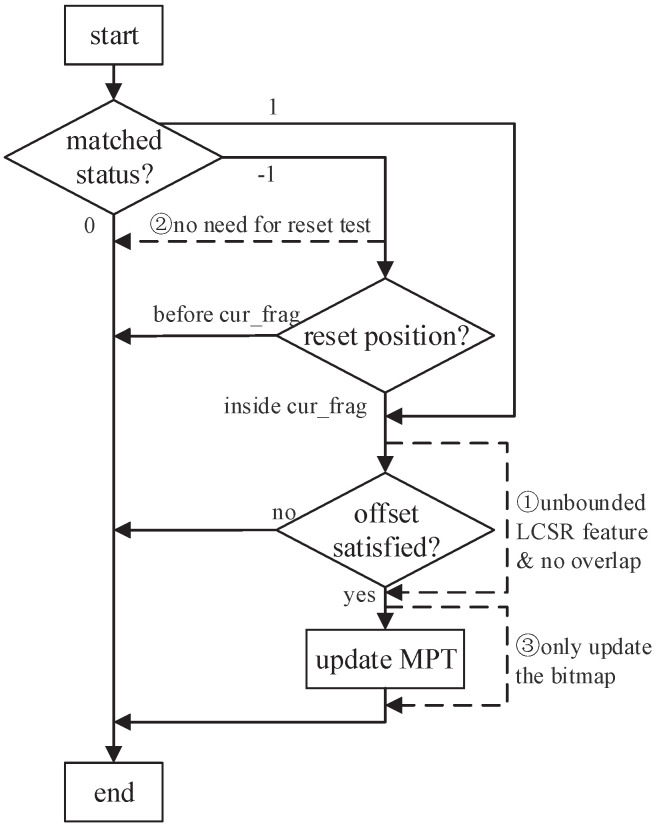
Optimization for the matching process; dotted lines with numeric labels are the optimized bypass procedure.

Similarly, for $frag_{1}$[ˆc]*$frag_{2}$ features, if $frag_{1}$ is confirmed as a valid match, and $frag_{1}$ and $frag_{2}$ have no overlap, then there is also no need to check the offset condition when $frag_{2}$ is matched. As no overlap exists between $frag_{1}$ and $frag_{2}$, if they are matched sequentially, the matching path of $frag_{2}$ cannot be started until $frag_{1}$ is matched. Combined with the first conclusion, we can add a bypass to the matching process to avoid the unnecessary offset condition test in the above situations. The dotted line labeled with ① in Figure 10 denotes the corresponding bypass operation.

Second, for $frag_{1}$[ˆc]*$frag_{2}$ features, if excluded character *c* does not appear in $frag_{2}$, we can also perform similar optimizations for the matching validity of $frag_{1}$. If fragment $frag_{2}$ is matched, and the matching status of $frag_{1}$ is −1, there is no need to check the matching validity of $frag_{1}$. Because characters from $frag_{2}$ can never reset fragment $frag_{1}$, the matching of $frag_{1}$ is invalid, and there is no need for further MPT update. Similarly, a bypass procedure (labeled with ② in Figure 10) is added to avoid the unnecessary reset validation test.

With the two bypass optimizations above, the offset-condition and reset-validation tests are executed only when needed. Unbounded ‘.*’ features and ‘[ˆc]*’ features account for the majority of the LCSR features in practice, and only a tiny proportion of unbounded LCSR features among them need a further offset-condition or reset-condition validation. Thus, the above two bypass operations apply to the vast majority of the fragments during matching, and many read operations and computational operations upon the MPT can be saved. Additionally, the bypass condition can be automatically identified in the compilation process. Further, it is encoded in the corresponding fragment labels (implementation details are discussed in Section 5). Thus, the judgment of bypass conditions would not bring any extra cost during matching.

## 5. Implementation Details

In this section, we provide the implementation details of Offset-FA. As the generation and matching algorithms were discussed in Section 3, here, we mainly discuss the main data structures for implementation. The data structure specifically includes the static structure of Offset-FA and the dynamic structure for matching (the MPT). To achieve good matching performance, the structures should be compact enough and easy for fast access.

**Figure 11:**
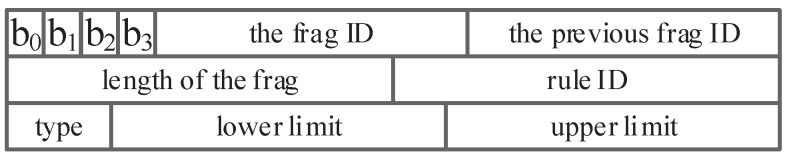
Organization for the fragment information structure.

Offset-FA comprises three parts: Fragment-DFA, the fragment relation table, and the reset table. Fragment-DFA can be stored as a two-dimensional array, and the reset table can be stored as a standard list. Both can be accessed efficiently. Thus, here, we mainly discuss the organization of the fragment relation table. When an accepted state in Fragment-DFA is activated, the matching engine returns a fragment information structure, as shown in Figure 11, rather than just the corresponding fragment ID. The fragment information structure includes all the necessary information about the matched fragment for further processing. It is composed of three 32-bit data. As they are stored contiguously and can be read out sequentially from the DDR3 memory, the time cost for reading the fragment information structure can be counted as one memory access.

In the fragment information structure, the $b_{0}$ bit indicates whether reset condition validation is needed if the matching status of the previous fragment is −1, and $b_{1}$ bit represents whether the offset condition test is required if the previous fragment is confirmed to be matched. As we discussed in Section 4.2, for most fragments, there is no need to further execute the reset-validation and offset-condition tests; thus, the $b_{0}$ and $b_{1}$ bits are usually set as 0. The $b_{2}$ bit denotes whether this fragment is the last in its corresponding pattern, and the $b_{3}$ bit is introduced later. The following information includes the matched fragment ID field, its previous fragment ID field, the length field of the matched fragment, and the related rule ID field (if the matched fragment is the last in the original pattern). The last 32 bits denote the corresponding offset range information; among them, the first 2 bits (named the type field) represent the four kinds of interval types, namely, ‘[]’, ‘[)’, ‘(]’, ‘()’.

In fact, as the last two 32 bits in the fragment information structure are unnecessary for most fragments, we could employ another optional storage strategy. The corresponding rule IDs are separately organized as a list, and the engine only stores the first 32 bits of the fragment information structure for fragments whose $b_{0}$$b_{1}$ value is 00. This strategy would not reduce the time cost for achieving the fragment information because it also requires one memory access for each fragment, but it could reduce the space cost to some extent.

The matching process table is organized into two components: the matching status and match/reset position. The matching status of all fragments can be recorded as a bitmap, and each fragment occupies two bits to represent the three kinds of matching status (0, 1, and −1). As this bitmap is compact enough, it can be deployed on fast on-chip memories, such as the caches. The match/reset position part for fragments can be organized as a list, and each item occupies 32 bits (16 bits for the match position and the other 16 bits for the reset position). Thus, the matching information about the previous fragment can be achieved with only one memory access. In fact, with the matching optimizations in Section 4.2, there is no need to read the match/reset position item for most fragments.

On the basis of the above data structures, we can analyze the time cost for processing a matched fragment. When a fragment is matched in Fragment-DFA, a read operation is sent to obtain the corresponding fragment information, including the previous fragment ID, fragment length, and offset range. Further, supposing that the matching status of the previous fragment is not 0, another read operation is sent to obtain the match/reset position item of the previous fragment only if one of the following two conditions is fulfilled. The matching status of the previous fragment is −1 and the $b_{0}$ bit of the matched fragment is 1, or the matching status of the previous fragment is 1 and the $b_{1}$ bit of the matched fragment is 1.

Lastly, if the matching of the current fragment is confirmed, a write operation is sent to record the matching position of the current matched fragment. In fact, this write operation is also redundant for most fragments. The purpose of a fragment’s match/reset position is to confirm the matching of its following adjacent fragment through the reset condition test or offset condition test. However, both the $b_{0}$ and $b_{1}$ bits are 0 for most fragments, which means that the matching confirmation for these fragments does not rely on the match/reset positions of their previous fragments. Thus, most fragments do not need to record the match/reset items. On the basis of the above analysis, we can further optimize: if the $b_{0}$$b_{1}$ value of a fragment is 00, the MPT does not record the match/reset position item for its previous fragment. We could employ the $b_{3}$ bit from the fragment information structure to denote whether the MPT records its match/reset positions, thus avoiding the unnecessary write operation when this fragment is confirmed as matched. For this kind of fragment, if it is confirmed as matched, we only need to update the matching status bitmap. Thus, we can add another bypass labeled with ③ in Figure 10 to avoid updating the match/reset items. Further, for a fast and efficient MPT operation, a renumbering method is applied to assign continuous IDs to the fragments with match/reset items.

As the time cost of reading the bitmap can be neglected, in the worst case, fragment processing requires three memory accesses (one read operation on FRT, one read operation on MPT, and one write operation on MPT). In practice, the three bypass optimizations apply to most situations. Thus, for most fragments, only the read operation on FRT is involved during matching.

## 6. Experimental Evaluation

In this section, we use various public regex rule sets and traces to verify the superiority of the Offset-FA method.

### 6.1. Experiment Setup

**Platform:** All experiments were conducted on an Intel Xeon CPU E5-2630 network server (CPU: 2.3 GHz, L1d cache: 32 KB, L2 cache: 256 KB, L3 cache: 15,360 KB, Mem: 32 GB, OS: CentOS 6.3).

**Rule sets:** In our evaluation, we tested 4 public regex rule sets. Table 3 summarizes the main characteristics of these rule sets. The first rule set comes from the Linux application protocol classifier, the second rule set is from the Bro NIDS, and the last two sets are from the Snort NIDS. The backdoor and spyware-put files contain many pure string patterns. To better test the applicability on regular expression patterns, we only extracted the RE patterns from these files. The second column denotes the number of rules in each rule set, and the last two columns list show the unbounded and bounded *LCSR* features that each rule set contains. In fact, these rule sets have many more closures and counting repetitions than those listed. We only counted the explosive *LCSR* features with the guidelines from Section 4. Features such as ‘∖s+’, ‘[a-zA-Z0-9]*’ and ‘[0-9A-z]{4}’ were not counted because they incurred limited state expansion.

**Traces:** We used the public traces from MIT Lincoln Lab [48] for performance evaluation. The traces contained 28 Internet tcpdump files from 146 to 748 MB. For each rule set, we randomly selected 5 trace files for matching, and computed the average memory bandwidth requirement as the matching speed.

**Evaluation metrics:** Construction time, space cost for automata, and memory bandwidth requirement. The semantic equivalence was also verified through experiments.

**Solutions to be compared:** Almost all the software solutions find a trade-off between memory cost and matching speed, and NFA and DFA are the two extreme solutions; they are referred to as the baseline in our comparison. Due to the fast matching speed of DFA, most state-of-the-art alternatives focus on DFA compression technologies. However, none of the tested rule sets could be built to a single DFA on our platform; thus, these compression solutions could not be implemented, as the original DFA was not available. In fact, as our Offset-FA proposal is orthogonal to the compression technologies, all existing compression algorithms could be applied to the Fragment-DFA part of Offset-FA.

MFA [45] cannot handle large bounded LCSR features and false positive/negative situations for the unbounded LCSR features in these rule sets. For the remaining solutions, the hybrid FA [37] and multiple DFAs [34] were the most practical to handle the state explosion problem. On the other hand, they can be generated automatically and keep semantic equivalence with the original rules. Thus, the hybrid FA [37] and multiple DFAs [34] were selected as the leading solutions for comparison.

### 6.2. Comparisons with State-of-the-Art Alternatives

**Offset-FA analysis:**Table 4 and Table 5 summarize the main characteristics when compiled to Offset-FA and other automata. Columns 2 to 5 in Table 5 describe the main components of the corresponding Offset-FA, including the number of split fragments, the number of states in Fragment-NFA, and the number of reset items. None of these rule sets could be compiled into a single DFA on our platform with 32 GB memory, which can store 32 million DFA states. Apparently, the rule sets are complex enough even though their rule numbers seem insignificant. The Bro set was the simplest; though it had only 7 unbounded *LCSR* features and 4 bounded *LCSR* features, the state explosion was still unacceptable. The single rule (SEARCH ∖/HTTP∖/1∖.1∖x0d∖x0aHost∖x3a.{0,251}∖x0d∖x0a∖x0d) with bounded *LCSR* feature ‘.{0,251}’ in Bro could generate more than 30 million states, not to mention the interaction with the unbounded *LCSR* features from other rules when compiled together.

On the other hand, Offset-FA obviously solved the state explosion problem very well by detaching explosive *LCSR* features. The numbers of fragment-DFA states in Bro and spyware-put were comparable to the numbers of corresponding NFA states. The most explosive situation occurred in L7, where the state number of fragment-DFA was about 20 times of the corresponding NFA’s state number. Because we employed strict standards to split the rules, many features such as ‘[∖x09-∖x0d -∼]*’ were not regarded to be *LCSR* features even they would cause a certain level of state expansion. However, compared with other solutions, the state expansion was much more limited and controllable in our Offset-FA because the most explosive *LCSR* features, such as ‘.*’, ‘[ˆ∖n]*’, and ‘.{200}’, were detached from the fragments.

**Figure 12:**
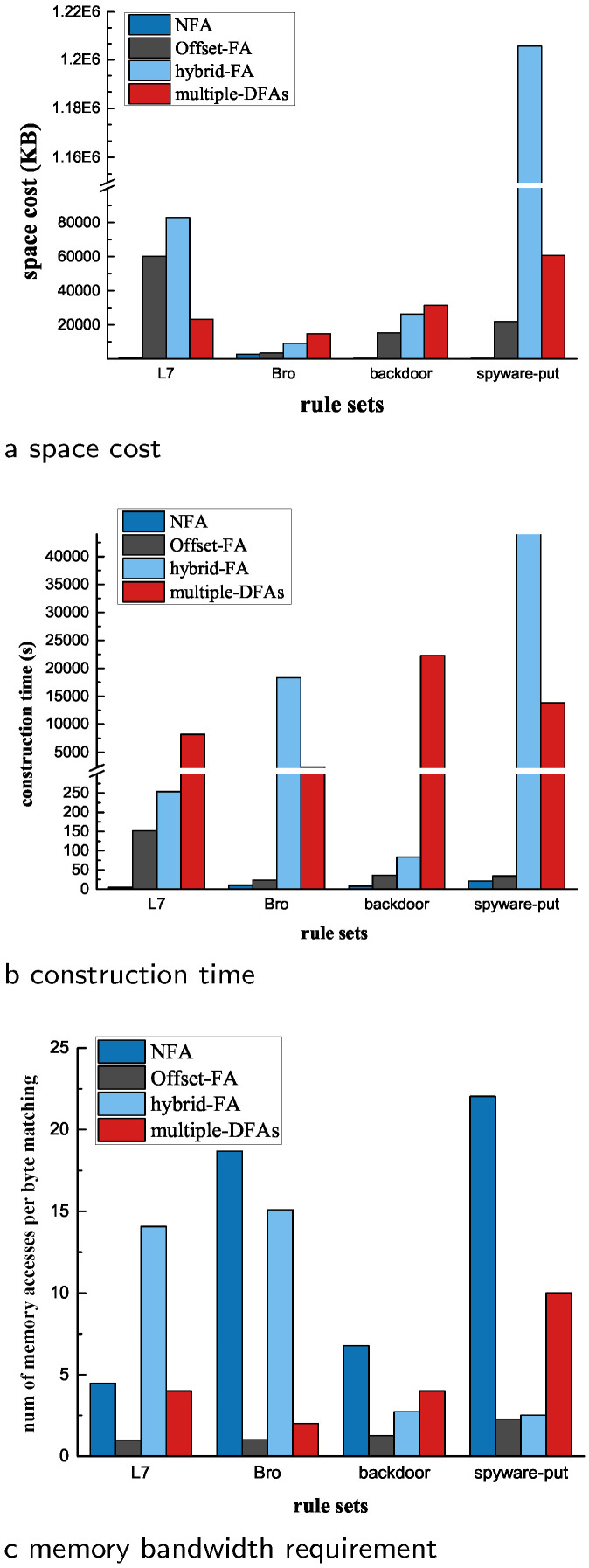
Space cost, construction time, and memory bandwidth requirement for these solutions.

**Space cost:**Figure 12a shows the space cost in different solutions. Obviously, NFA was the most space-efficient solution, partly due to the small state number of NFA. The other important factor is that, for most NFA states, only several labeled transitions need to be recorded, whereas all 256 transitions need to be stored for other DFA-like solutions. This is why the state number of Offset-FA was similar to the number of corresponding NFA states in the spyware-put set, but its space cost was 40 times larger than NFA.

Among the three other solutions, Offset-FA performed the best in almost all situations. The space cost of multiple DFAs was comparable with that of our Offset-FA in all rule sets, but its compilation time was huge and unacceptable. Moreover, multiple DFAs could not handle rules with large unbounded *LCSR* features. To generate multiple DFAs for the Bro set, we removed three rules (these rules had large unbounded *LCSR* features of ‘[ˆ∖x0A]{512}’, ‘.{0,251}’, ‘[ˆ∖x0A]{50}’, separately) with large bounded LCSR features, where each rule could generate more than 1 million states when compiled into a DFA. The hybrid FA performed better than multiple DFAs in the Bro and backdoor sets, and worse than multiple DFAs in the L7 and the spyware-put sets. Because more *LCSR* features are located at the head of patterns, these explosive features were compiled into the DFA part in hybrid FA. Especially in the spyware-put set, its space cost was about two orders of magnitude higher than that of Offset-FA and multiple DFAs.

**Construction time:** These solutions behave much more differently in construction time, as shown in Figure 12b. The time cost of NFA is the least in all rule sets because it avoids the time-cost NFA-to-DFA procedure. Among other solutions, Offset-FA was the most comparable with NFA. Though Offset-FA mostly avoids state expansion, NFA-into-DFA conversion still introduced much extra time cost. The most significant time cost of Offset-FA was 152 s, which occurred in the L7 set, but it is still a reasonable cost in practice.

On the other hand, the hybrid FA and Offset-FA had a huge time cost for the compilation procedure. Their time cost was about 1 to 3 orders higher than that of NFA, and the hybrid FA cost more than 12 h for the spyware-put set. In general, the time cost of the hybrid FA was linear with the number of head-DFA states. In the Bro set, the NFA reduction procedure also greatly contributed to the compilation time in hybrid-FA, especially when the rule set contained some large bounded *LCSR* features. The state number of multiple DFAs is not so large as that of the hybrid FA, but it still costs hours to compile these rule sets. The leading cause is that it needs to compute the relationship between every two rules and try many grouping combinations.

**Matching speed:**Figure 12c provides the average memory bandwidth requirement, namely, the average memory access for each byte processing. Offset-FA obviously outperformed other solutions in all rule sets. The average memory bandwidth requirement was under 2 in Offset-FA for the L7, Bro, and backdoor rule sets. Even for the most complex spyware-put set, the average memory access for each byte processing was still about 2.5. Considering the limited space cost of Offset-FA, it could achieve outstanding matching performance in practice, and an excellent trade-off between space cost and matching speed. The memory access in Offset-FA matching is mainly composed of two parts, state traversal in Fragment-DFA and the update operation for the matching process table. The first item is fixed and requires one memory access for each byte, whereas the second part is invoked only when a fragment is matched. Thus, the memory bandwidth requirement is determined by the update frequency of the operation is invoked and the amounts of memory are required for each update operation.

In fact, to achieve better matching speed, we sacrificed more space cost in the compilation procedure. First, the rules were split into longer rather than shorter fragments. Longer fragments mean that they have a lower probability of being matched, thus reducing the frequency with which fragments are matched. Second, with the strict definition of a large character set, reset symbols and items were highly reduced, which means the time cost for reset operations was also highly reduced. There is a little increase in memory access from the L7 to the spyware-put set that can be ascribed to the increased number of reset items (from 0 to 499).

**Figure 13:**
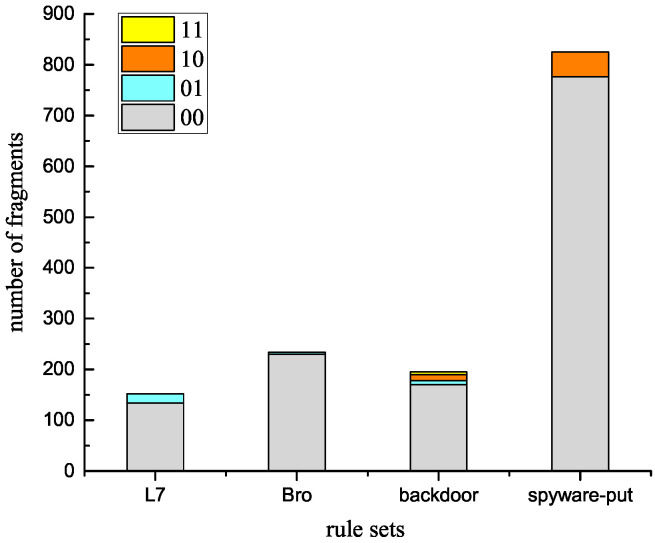
Statistic of fragments in each rule set classified with their $b_{0}$$b_{1}$ values.

On the other hand, optimizations in Section 4 and Section 5 also greatly contributed to reducing the number of memory accesses when fragments are matched.Figure 13 provides the number of each kind of fragment in these rule sets according to their $b_{0}$$b_{1}$ values. Fragments with a $b_{0}$$b_{1}$ value of 00 occupied the vast majority (from 87% to 98%) in each rule set. This means that, when these fragments were matched in Fragment-DFA, there was no need to check the match/reset position items of their previous fragments (corresponding to Optimizations ① and ②). The matching engine only consumed one memory access to obtain the ID of the previous fragment, then checks the matching status of the previous fragment from the bitmap to confirm the matching of the current fragment. Further, for fragments with a $b_{0}$$b_{1}$ value of 00, as the confirmation of these matching does not rely on the match/reset positions of their previous fragment, the matching engine would not record the match/reset position items for their previous fragments. Accordingly, their previous fragments would not involve any update operations on the match/reset position field, and the matching engine only needed to update the bitmap part for them (corresponding to Optimization ③).

The above optimizations would largely reduce the number of matched fragments and the number of memory accesses when fragments are matched. Thus, the time cost for the MPT update part is significantly reduced, which greatly contributes to the overall matching performance.

Further, neither the L7 nor the Bro set had 10- or 11-type fragments, which means that none of the fragments in these two sets needed the reset validation test when matched (corresponding to Optimization ①). Similarly, none of the fragments in the spyware-put set needed the offset condition test when matched (corresponding to Optimization ②). For all the rule sets, only 5 fragments required both the reset-validation and the offset-condition tests, and they all appeared in the backdoor set.

NFA undoubtedly performed the worst, as it required almost 22 memory accesses for each byte processing in the spyware-put set. The performance of the hybrid FA was very volatile and required even more memory accesses than NFA did for the L7 set because the border states were activated frequently, and their corresponding tail NFA states had to be handled sequentially. For a given upper limit of a single DFA in the multiple-DFA solution, the number of DFAs increased linearly with the scale and complexity of the rule sets, and the memory bandwidth requirement. Thus, scalability for the multiple-DFA solution is challenging.

## 7. Conclusions

In recent years, with the rapid development of information technology, CPS has penetrated all aspects of our lives, and CPS security has become a vital issue that cannot be ignored. Signature-based intrusion detection is the basis of CPS security protection, and this paper mainly studied the state explosion problem faced by regular expression matching. The main goal of regular expression matching is to achieve a reasonable matching speed under moderate platforms, and the main challenge is the state explosion problem. Most researchers are devoted to exploiting solutions leading to a good trade-off between space cost and matching speed, but state explosion remains a great challenge in practice.

In this paper, we attributed the state explosion problem to features of large character sets with unbounded (closures) or bounded (counting) repetitions, which we call *LCSR* features. A novel automaton, Offset-FA, was devised to handle such features. In our Offset-FA, these explosive *LCSR* features are detached from original rules and are then represented as tables to omit severe state expansion while keeping semantic equivalence. With the separation of *LCSR* features, the state explosion problem is well-handled. The space cost of Offset-FA outperformed that of state-of-the-art solutions in practical rule sets and was comparable with NFA in some situations. On the other hand, with a well-designed matching mechanism and a series of efficient optimizations, Offset-FA can provide fast matching speed, and the average memory bandwidth requirement can be limited to about 2 memory accesses for each byte processing. To our knowledge, Offset-FA achieves the best trade-off between space cost and matching speed, and outperforms other state-of-the-art solutions to state explosion.

## Figures and Tables

**Table 1:** Time and space complexity comparisons of NFA and DFA in various strategies.

	Compile *m* REs into *m* FAs		Compile *m* REs into an Integrated FA	
	Processing Complexity	Storage Cost	Processing Complexity	Storage Cost
NFA	$O\;(mn^{2})$	O (mn)	$O\;(mn^{2})$	O (mn)
DFA	O (m)	$O\;(m2^{n})$	O (1)	$O\;(2^{mn})$

**Table 2:** Configuration of fragment relation table for general *LCSR* features.

Various Features	Offset Range for ${frag}_{i+1}$
$frag_{i}.*frag_{i+1}$	[Len${}_{frag_{i+1}}$,)
$frag_{i}.+frag_{i+1}$	[Len${}_{frag_{i+1}}$+1,)
$frag_{i}$[ˆc]*$frag_{i+1}$	[Len${}_{frag_{i+1}}$,)
$frag_{i}$[ˆc]+$frag_{i+1}$	[Len${}_{frag_{i+1}}$+1,)
$frag_{i}.\left\{n\right\}frag_{i+1}$	Len${}_{frag_{i+1}}$+*n*
$frag_{i}.\left\{m,n\right\}frag_{i+1}$	[Len${}_{frag_{i+1}}+m$,Len${}_{frag_{i+1}}$+*n*]
$frag_{i}.\left\{,m\right\}frag_{i+1}$	[Len${}_{frag_{i+1}}$,Len${}_{frag_{i+1}}$+*m*]
$frag_{i}.\left\{n,\right\}frag_{i+1}$	[Len${}_{frag_{i+1}}$+*n*,)
$frag_{i}$[ˆc]$\left\{n\right\}frag_{i+1}$	Len${}_{frag_{i+1}}$+*n*
$frag_{i}$[ˆc]$\left\{m,n\right\}frag_{i+1}$	[Len${}_{frag_{i+1}}$+*m*,Len${}_{frag_{i+1}}$+*n*]
$frag_{i}$[ˆc]$\left\{,m\right\}frag_{i+1}$	[Len${}_{frag_{i+1}}$,Len${}_{frag_{i+1}}$+*m*]
$frag_{i}$[ˆc]$\left\{n,\right\}frag_{i+1}$	[Len${}_{frag_{i+1}}$+*n*,)

**Table 3:** Tested rule sets and their features.

Rule Sets	Rules	LCSR Features	
		Unbounded	Bounded
L7	95	29	16
Bro	226	7	4
backdoor	126	65	10
spyware-put	414	411	0

**Table 4:** Characteristics of NFA, DFA, hybrid-FA, and multiple DFAs for the datasets in Table 3.

Rule Sets	NFA	DFA	Hybrid-FA				Multiple-DFAs	
	States	States	Head-DFA	Tails	Tail-NFA	Border States	DFAs	States
L7	3071	>32 M	89,494	32	862	41,271	4	23,323
Bro	3857	>32 M	6550	11	963	30	2	14,896
backdoor	3939	>32 M	25,896	32	661	363	4	31,492
spyware-put	12,446	>32 M	5,399,048	43	4871	39,968	10	60,598

**Table 5:** Characteristics of Offset-FA for the datasets in Table 3.

Rule Sets	Offset-FA			
	Frags	Frag-NFA	Frag-DFA	Reset Items
L7	152	2854	60,107	0
Bro	234	3020	3536	2
backdoor	195	3790	15,389	86
spyware-put	825	11,810	22,013	499

## Data Availability

Not applicable.
